# Enhancement of the Sensing Performance of Devices based on Multistimuli-Responsive Hybrid Materials

**DOI:** 10.1021/acsami.3c08376

**Published:** 2023-09-13

**Authors:** Taher Abu Ali, Marlene Anzengruber, Katrin Unger, Barbara Stadlober, Anna Maria Coclite

**Affiliations:** † Graz University of Technology, NAWI Graz, Institute of Solid State Physics, 8010 Graz, Austria; ‡ Joanneum Research Forschungsgesellschaft mbH, MATERIALS − Institute for Surface Technologies and Photonics, 8160 Weiz, Austria; § Electronic Sensors, Silicon Austria Laboratories GmbH, 8010 Graz, Austria

**Keywords:** multichamber reactor, stimuli-responsive, hydrogel, piezoelectric, core−shell nanostructures

## Abstract

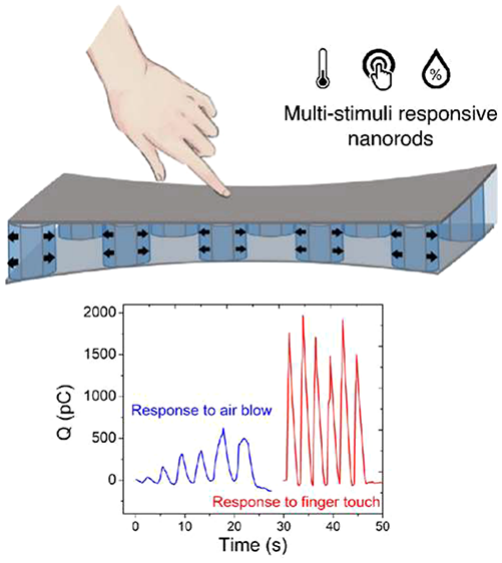

Capturing environmental stimuli is an essential aspect of electronic skin applications in robotics and prosthetics. Sensors made of temperature- and humidity-responsive hydrogel and piezoelectric zinc oxide (ZnO) core–shell nanorods have shown the necessary sensitivity. This is achieved by using highly conformal and substrate independent deposition methods for the ZnO and the hydrogel, i.e., plasma enhanced atomic layer deposition (PEALD) and initiated chemical vapor deposition (iCVD). In this work, we demonstrate that the use of a multichamber reactor enables performing PEALD and iCVD, sequentially, without breaking the vacuum. The sequential deposition of uniform as well as conformal thin films responsive to force, temperature, and humidity improved the deposition time and quality significantly. Proper interlayer adhesion could be achieved via in situ interface activation, a procedure easily realizable in this unique multichamber reactor. Beyond the fabrication method, also the mechanical properties of the template used to embed the core–shell nanorods and the cross-linker density in the hydrogel were optimized following the results of finite element models. Finally, galvanostatic electrochemical impedance spectroscopy measurements showed how temperature and humidity stimuli have different effects on the device impedance and phase, and these differences can be the basis for stimuli recognition.

## Introduction

The performance of sensor materials is commonly evaluated by their response toward external stimuli: a large amplitude and a fast response upon excitation are desirable. Lately, hydrogels have attracted growing interest for the integration in smart devices due to their unique properties, such as elasticity, transparency, biocompatibility, and the ability to conduct ions due to the presence of water molecules.^1−6^ Hydrogels are three-dimensional polymer networks with the ability to incorporate water into their structure and even double or triple their initial volume when immersed in water or exposed to water vapors.^7,8^ Stimuli-responsive hydrogels exhibit significant change in their properties (e.g., volume, index of refraction, wettability) when exposed to an external stimulus like humidity, temperature, light,^9^ pH,^10^ or magnetic and electric field.^11^ For temperature-responsive hydrogels, a lower critical solution temperature (LCST) can be observed.^12^ At this temperature, a phase change takes place, and the hydrogel network collapses from its expanded state into a globule state as the temperature is increased.^13^ The strong volume change stemming from the sharp LCST transition can be easily detected and converted into an electrical output with the use of electrical transduction, and therefore, it is extremely useful for sensors to achieve large signal amplitude and a fast response. Hydrogels based on biocompatible polymers with an LCST in the physiological temperature range are of particular interest for applications in tissue engineering,^14,15^ biotechnology,^16^ or wearable electronics.^17^

**Figure 1:**
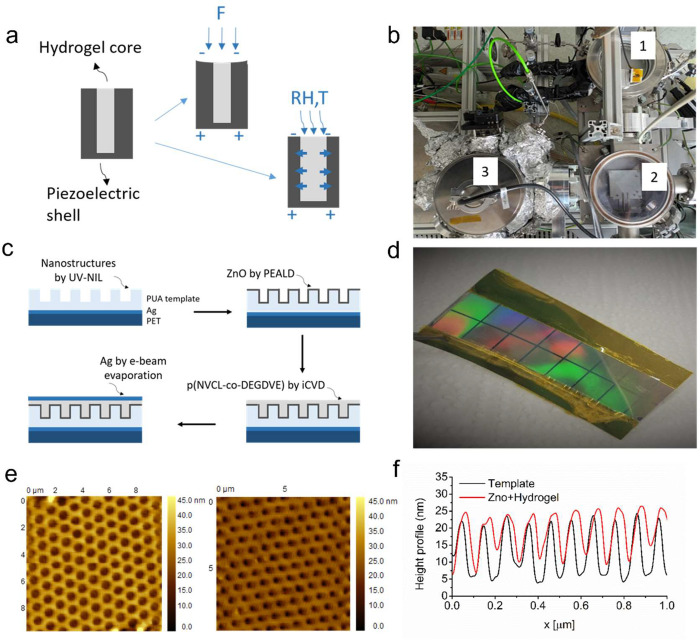
(a) Schematics of a multistimuli-responsive core–shell nanorod explaining the detection concept and responsiveness to force (directly sensed by ZnO shell), humidity, and temperature (swelling of p(NVCL-*co*-DEGDVE) core is translated to piezoelectric response through stress/strain). (b) Combined reactor, which consists of (1) iCVD reactor, (2) Transfer chamber, (3) PEALD reactor. (c) Multistimuli-responsive nanostructured sensor array fabrication steps. Dimensions are not indicated to scale. (d) An image of the full device prior to deposition of the Ag top electrodes. The colors come from the diffraction grating of the template in the visible spectrum. (e) 2D AFM topography image of the nanostructured PUA template prior to filling (left) and after partial filling of the nanotrenches with 50 nm of ZnO and 150 nm of hydrogel (right). Both clearly show periodic nanotrenches with hexagonal grid arrangement. (f) Line scan of the AFM images of before (black) and after (red) partial filling of the nanotrenches.

In a previous work, we successfully combined a piezoelectric semiconductor material, namely, zinc oxide (ZnO), and a multistimuli-responsive hydrogel, namely poly(*N*-vinylcaprolactam-*co*-di(ethylene glycol) divinyl ether) or p(NVCL-*co*-DEGDVE) in core–shell nanorod structures capable of detecting force, humidity, and temperature. As depicted in Figure 1a, the stimuli detection is achieved through measuring the piezoelectric current generated upon deformation of the ZnO shell due to the hydrogel core swelling in response to humidity (max sensitivity *S*_*H*_ = 1.2 nC %^–1^ to relative humidity (RH) in the range of 85–96% at 25 °C) and temperature (max sensitivity *S*_*T*_ = 0.14 nC °C^1–^ response in the range 30–50 °C at 96% RH).^17^ Additionally, the piezoelectric properties of ZnO allow direct detection of the applied force (max sensitivity *S*_*F*_ = 36 pC N^1–^), with site-specific force sensing and a resolution down to 0.25 mm^2^.^17^ The fabrication of such core–shell nanorod structures requires a deposition process able to operate at low temperatures due to the nature of the hydrogel and the template material. Additionally, highly conformal thin films are required for the realization of a nanostructured geometry. Initiated chemical vapor deposition (iCVD) is an ideal deposition method for polymer thin films due to its substrate independence and the wide range of possible reactants. Under certain conditions, the conformality of the process allows for the low temperature deposition of high quality polymer films even on substrates with complex geometries.^17,18^

Plasma enhanced atomic layer deposition (PEALD) allows to deposit metal oxides at low temperature, with high conformality to three-dimensional substrates.^19^ PEALD can be operated at low temperatures, since the reactions are plasma-driven instead of thermally activated.^20,21^ This makes PEALD the perfect candidate for coating temperature sensitive substrates. Combined with iCVD, the fabrication of uniform and conformal high quality hybrid thin films can be realized. Until now, the fabrication of a thin film consisting of one layer deposited via PEALD and a second deposited via iCVD was possible only with the consecutive layer fabrication in two separate reactors. This is a rather lengthy process, and since the interface between the two layers is exposed to ambient conditions during the transfer from one chamber to the other, contamination can be expected to some extent. A combination of both deposition systems in a single multichamber reactor would therefore hold benefits like the reduction of overall deposition time and the simultaneous improvement of the multilayer quality.

In this work, we performed the deposition of structured core–shell thin films in a one-of-a-kind multichamber reactor combining PEALD and iCVD and showed the advantages of interface optimization on the sensing properties. Additionally, the work presents steps toward performance enhancement, namely, the influence of the template material mechanical properties and the p(NVCL-*co*-DEGDVE) cross-linker (CL) fraction. Finally, characterization with varying humidity and temperature using galvanostatic electrochemical impedance spectroscopy (GEIS) is presented as an additional method for signal readout, which in combination with the piezoelectricity measurements could lead to stimuli recognition.

## Experimental Section

### Sensor Fabrication

Multistimuli-responsive nanostructured multilayer thin films were fabricated using a custom-built reactor consisting of a PEALD chamber connected via transfer chamber to an iCVD chamber, as shown in Figure 1b. This enabled the deposition of 200 nm of p(NVCL-*co*-DEGDVE) on top of the 50 nm of the ZnO layer without breaking the vacuum. To structure such films and obtain core–shell nanorods, we used templates made by UV nanoimprint lithography (UV-NIL). With this technique, two polyurethane acrylate resins (PUA, NILcure, Joanneum Research, Austria), with different mechanical properties (Young’s modulus, *E*_*hard*_ = 2 GPa and *E*_*soft*_ = 200 MPa), were nanostructured on top of a silver (Ag) coated polyethylene terephthalate (PET) substrate (50 μm, Hueck Folien, Austria). The resultant nanotrenches had a diameter *d* = 500 nm, height *H* = 500 nm, aspect ratio *AR* = 1, and a pitch = 1000 nm and were arranged in 16 square fields each with dimensions of 8 × 8 mm. More details on the setup can be found elsewhere.^17^ The samples were then placed in the transfer chamber, with ∼20 μbar of pressure, and transferred into the PEALD chamber for the first deposition step. Afterward, the samples were transported to the iCVD chamber without breaking the vacuum for the second deposition step.

The stainless-steel PEALD chamber had a volume of 5.28 L with an inner diameter of 100 mm and a height of 137.8 mm. Via a stainless-steel transfer boat (70 × 70 mm), the samples were placed on the heated (35 °C) sample stage, made from pyrolytic boron nitride (Boratec). The distance between the ground and the RF top electrode was 78 mm. A gate valve (VAT, Switzerland) regulated the sample transfer between the two chambers. A two-stage rotary vane pump (DUO 20 Pfeiffer Vacuum, Germany) and a turbopump (Pfeiffer Vacuum, Germany) maintained a working pressure of 200 μbar and were connected to the PEALD chamber, where the pressure was controlled via a butterfly throttle valve (MKS Instruments, USA). Further, a Baratron Type 626 pressure transducer and a PDR2000A (Two-Channel Digital Power Supply and Readout, MKS Instruments, USA) were employed for the readout. Purging gas and coreactant flows were controlled via the GE50A mass flow controller (MKS Instruments, USA). 60 W plasma was generated via the Cesar RF power generator (Advanced Energy, USA) connected to a matching network (Navio, Advanced Energy, USA). The deposition process consisted of four steps: 15 s O_2_ plasma dose, 15 s argon purge, diethylzinc (DEZ, Sigma-Aldrich, USA) dose, and a second 15 s argon purge. This deposition recipe was adapted from the one previously optimized in our group.^22,23^ A 0.15 s pulse, controlled via an ALD valve (Swagelock ALD3, USA), introduced DEZ into the chamber. The desired thickness (50 nm) was reached after 250 cycles, and the deposition was finalized with a plasma step to activate the surface for the following deposition.

The volume of the iCVD chamber was 3.15 L. A recirculating chiller (Polar Series 500 LC, Thermo Scientific, USA) was connected to the aluminum sample stage (*d* = 85 mm and *H* = 35 mm). A two-stage rotary vane pump (DUO 20, Pfeiffer Vacuum, Germany) protected via zeolite filter (Molecular Sieve Foreline Trap, Kurt J. Lesker Company, USA) was connected to the reactor. The chamber pressure was monitored via a pressure controller (600 Series, MKS Instruments, USA) and regulated via a butterfly throttle valve (MKS Instruments, USA) and a manual valve (XLH, High Vacuum Manual Angle Valve, SMC, Japan). Between the monomer inlet and sample stage, a perforated diffuser plate was installed to ensure homogeneous gas mixing. The filament inside the chamber was heated resistively by a low voltage power supply (PTN 350-5, Heinzinger, Germany) to 200 °C.

p(NVCL-*co*-DEGDVE) was deposited directly on the ZnO. *N*-Vinylcaprolactam (NVCL, Sigma-Aldrich, 98% purity, USA) was used as a monomer, di(ethylene glycol) divinyl ether (DEGDVE, Sigma-Aldrich, 99% purity, USA) as cross-linker, and *tert*-butyl peroxide (TBPO, Sigma-Aldrich, 98% purity, USA) as initiator. All chemicals were used without further purification. The monomer and cross-linker jars were heated to 85 and 70 °C, respectively, and the line heating set to 90 °C, while the initiator jar and line remained at room temperature. Flow rates of 0.1 ± 0.05 sccm for NVCL, 0.1 ± 0.05 sccm for DEGDVE, and 0.9 ± 0.05 sccm for TBPO resulted in the formation of water-stable and highly responsive hydrogel thin films with ∼25% nominal cross-linking. Alternatively, a DEGDVE flow rate of 0.2 ± 0.03 sccm was used to obtain ∼35% nominal cross-linking. The deposition was run at a working pressure of 466 μbar and at 30 °C (substrate temperature). A laser interferometry setup consisting of a HeNe laser (λ = 632.8 nm, HNLS008L-EC, THORLABS, USA) and an energy meter (PM100USB, THORLABS, USA) was used for thickness monitoring of the hydrogel layer grown on a Si(100) wafer, which was positioned next to the structured template inside the reactor chamber. After the hydrogel deposition was completed, the samples were removed from the reactor. Silver (Ag, 50 nm), serving as a top electrode, was deposited via e-beam evaporation with a deposition rate between 0.1 and 0.2 nm s^–1^. Using a stainless-steel shadow mask, eight neighboring Ag electrode fields were deposited, each with an active area of 6 × 6 mm.

### Characterization

The nanostructured samples were investigated for conformality via atomic force microscopy (AFM, Nanosurf Easyscan 2, Switzerland) with a NANOSENSORS scanning probe (model PPP-NCLR-20). The topography of semifilled nanostructures and template material was recorded.

The piezoelectric response of the nanostructured multilayer samples was investigated in an in-house built setup with a step force-signal *F* = 10, 12, and 15 N and frequency *f* = 0.5 Hz. A detailed description of the setup can be found elsewhere.^17,20^

The generated piezoelectric charge was measured in a climate chamber (Espec SH222, interior volume 22 L, Japan) with a data acquisition system (DAQ, SIRIUS Multi, Dewesoft, Slovenia) in the conditions RH = 35–95% at *T* = 25 °C and *T* = 40 °C; otherwise, *T* = 10–50 °C at RH = 40%. More details on the setup can be found elsewhere.^20^ Additionally, an alternative custom-built setup was used. As described in ref (25), it varies the relative humidity from 5 to 75% by mixing N_2_ bubbled through water and pure N_2_ via needle valves at room temperature. For comparison with the simulated data and our previous work, the charge density σ was calculated by normalizing the charge to the electrode area.

The humidity and temperature response of the devices were investigated via galvanostatic electrochemical impedance spectroscopy (GEIS) executed with a two-electrode setup (Gamry 6000 Reference, USA) inside the same climate chamber. Measurements were performed within a frequency range *f* = 200–1500 Hz, where the AC current is set to 1 × 10^–7^ A and at three different temperatures: 10, 23, and 35 °C. The relative humidity was raised from 30 to 95% for each temperature in the environmental chamber mentioned above. The set points were controlled via Python scripts where an accuracy of ±0.2 °C and ±1% for temperature and RH, respectively, was defined. For comparison, GEIS measurements were also performed on samples obtained by directly depositing the hydrogel only, the ZnO only, and the hydrogel + ZnO layers on custom-made printed circuit boards (PCBs, Ni–Au coating, Eurocircuit, Germany), using for the depositions the same conditions and thicknesses as used in the full devices deposited on the PUA templates. The measurements were done in the 10 Hz–100 kHz frequency range with a resolution of 10 points per decade and by applying an AC voltage of 10 mV. These measurements were performed by varying the humidity in the range 5–85% at 25 °C and in a water bath at 10, 25, and 35 °C.

### FEM Simulations

COMSOL multiphysics v5.6 was utilized to perform an optimization study on the nanostructured multilayer thin films’ response to force, humidity, and temperature, using the piezoelectric multiphysics obtained from coupling of the solid mechanics and electrostatics modules. For this, a 3D model was developed to model the influence of the template material rigidity, namely the Young’s modulus *E* and the influence of the hydrogel cross-linker percentage. The geometry used within the 3D model is depicted in Figure S1. A 3D model of a cross-sectional single nanorod (*d*_*nanotrench*_ = 500 nm, height *H* = 500 nm, pitch = 1000 nm, *t*_*ZnO*_ = 50 nm, and *t*_*hydrogel*_ = 200 nm) was used, and multiple boundary conditions were applied, starting with a symmetry boundary condition applied to the front xz-plane and a periodic boundary condition applied to both yz-planes of the geometry. The hydrogel core was assigned as a hygroscopic material, and the swelling behavior in response to humidity (RH = 20, 30, 50, 60, 70, 80, 90, and 95%) and temperature (*T* = 10, 25, 35, and 50 °C) was modeled following eq S1. Laser interferometry was used to obtain the thickness change due to swelling, and dependently, the data was shown in Tables S1 and S2 with the procedure reported in previous work.^24^ The zinc oxide shell was modeled as single crystalline with preferential 002 orientation and it was assigned as a piezoelectric material following eqs S2 to S4. Previous XRD experiments have shown that ZnO obtained by PEALD at 35 °C is polycrystalline with some crystals oriented along the100 plane and some oriented along the 002 plane. Nevertheless, within the simulation, the piezoelectric polarization tensor is defined for bulk single crystalline ZnO. The PUA polymeric template was modeleld as a linear elastic material, with *E*_*soft,sim*_ = 200 MPa representing a soft polymeric template vs *E*_*hard,sim*_ = 2 GPa representing a hard polymeric template. A list of the material properties required for the model is given in Table S3. The top and bottom electrodes were simulated as floating and ground boundary conditions, respectively. Finally, a boundary load condition was applied to the top xy-plane of the geometry to simulate the input force *F* = 10, 12, 15, and 20 N.

## Results and Discussion

### Morphological Characterization

The fabrication steps elaborated in the Experimental Section are shown in Figure 1c. The resultant device prior to deposition of the top electrode is shown in Figure 1d. Kapton tape is applied to cover parts of the bottom electrode. During the electrical characterization, the Kapton tape is removed, exposing clean bottom electrode regions for contacting.

AFM topography images show the nanostructures prior to the deposition (Figure 1e) and after the deposition of ZnO and p(NVCL-*co*-DEGDVE) (Figure 1f) over a scan area of 10 × 10 μm^2^. The periodicity of nanostructures prior to filling clearly confirms the dimensions of the structures as well as the periodicity (*d* = 500 nm and pitch = 1000 nm, with a hexagonal grid arrangement). After partial filling of these nanotrenches with the core–shell nanorods (50 nm of ZnO and 150 nm of p(NVCL-*co*-DEGDVE)), no apparent change in the template periodicity could be observed (Figure 1e, right), which indicates the conformality of the PEALD and iCVD techniques. A line scan of the AFM images (Figure 1f) confirms the partial filling of the nanotrenches, since the full width at half maximum (fwhm) decreases from 502 nm for the PUA template to 322 nm. The line scan offers only information on the coverage of the upper 20 nm of the walls of the nanotrenches due to limitations of the AFM setup.

### Fabrication Method

**Figure 2:**
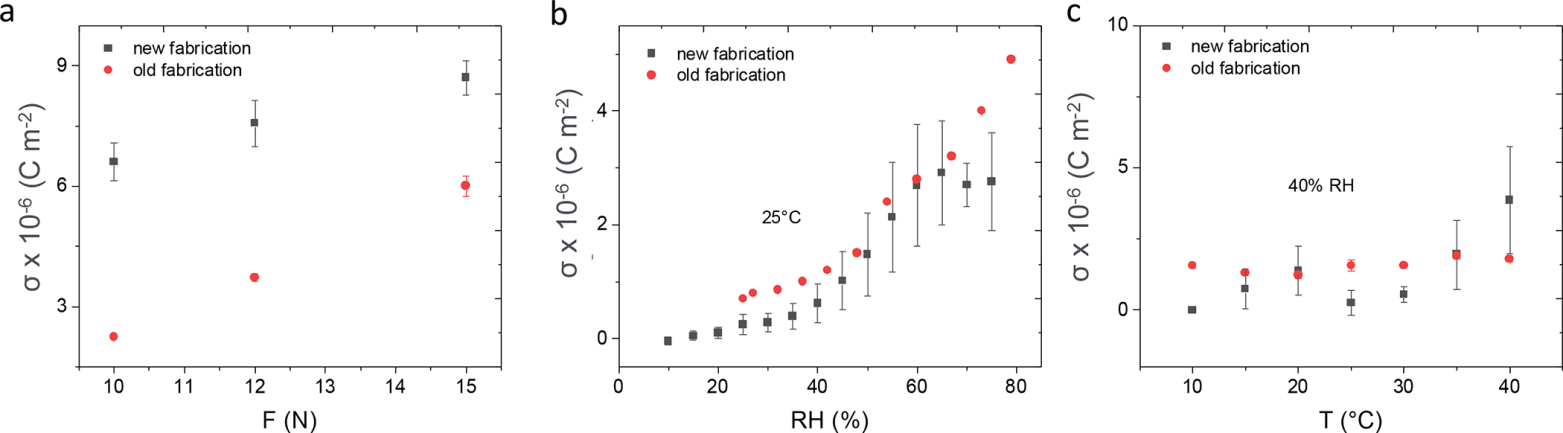
Piezoelectric charge density measured in response to force (a), over 8 to 10 cycles of press and release (the results are averaged over three electrode fields); to humidity (b) at 25 °C; and to temperature (c) at 40% RH on devices made with the old and the new fabrication setup. The standard deviations are reported as error bars on all data points, but in some cases, the bars are too small to be observed.

Figure 2 shows the piezoelectric characterization made on a device fabricated under the same conditions but with two different fabrication methods, i.e., one in which the deposition of the hydrogel and of the ZnO happens in separate reactors (named the “old fabrication method”) without interface activation and one in which the deposition of the two materials happens sequentially with interface activation (named the “new fabrication method”). The interface activation consisted of terminating the PEALD deposition with an O_2_ plasma pulse. It has been observed that this improves the adhesion of the subsequently deposited hydrogel to the ZnO surface. Without plasma exposure, the hydrogel would eventually delaminate from the ZnO upon exposure to water, whereas this was not observed if the ZnO surface was activated with an O_2_ plasma before hydrogel deposition. We conclude that the O* radicals, formed by the O_2_ plasma on the ZnO surface, lead to a better interface between the hydrogel and ZnO. Such activation was easily achievable in the dual reactor.

The devices were stimulated by force (Figure 2a), humidity (Figure 2b), and temperature (Figure 2c), and the corresponding piezoelectric charge density was measured.

Figure 2a shows the device piezoelectric response to step force signal *F* = 10, 12, and 15 N (*f* = 0.5 Hz) for 8 to 10 cycles of press and release, with the piezoelectric current *I* recorded as a function of time and the charge density σ (corresponding to the time integral of *I(t)* normalized by the electrode area), plotted as a function of *F*. It can be easily observed how the new fabrication route leads to an enhanced response (at *F* = 15 N and σ = 8.7 ± 0.4 × 10^–6^ C m^–2^ vs 6.1 ± 0.2 × 10^–6^ C m^–2^ for the old fabrication route). We believe that this could be due to the improved adhesion between the hydrogel and the ZnO that would ease the charge transfer from the top to bottom electrode during the piezoelectric measurements. Considering that the top electrode lays on the hydrogel layer, possible local delamination would cause charge dissipation at the interface. Figure 2b shows the piezoelectric response to humidity changes measured because of the swelling of the hydrogel core: when the hydrogel swells (upon water molecule intake), it exerts a stress on the ZnO shell, which translates into a piezoelectric charge output. The piezoelectric charge density is measured at 25 °C, i.e., below the LCST, considering that the LCST of the 25% cross-linked hydrogel was 34 ± 2 °C.^21^ The humidity response did not change significantly with the fabrication method.

Figure 2c depicts the response to a temperature profile (10–40 °C) at 40% RH. As shown in our previous contribution,^17^ the piezoelectric charge is constant at this humidity content, notwithstanding the change in temperature. The sensor fabricated with the new fabrication route, showed a maximum response of 4 × 10^–6^ C m^–2^ at 40 °C starting from 0 C m^–2^ at 10 °C. Meanwhile, the sensor fabricated with the old fabrication route showed a negligible change in the charge density for the same temperature range (σ = 1.5–2 × 10^–6^ C m^–2^).

**Figure 3:**
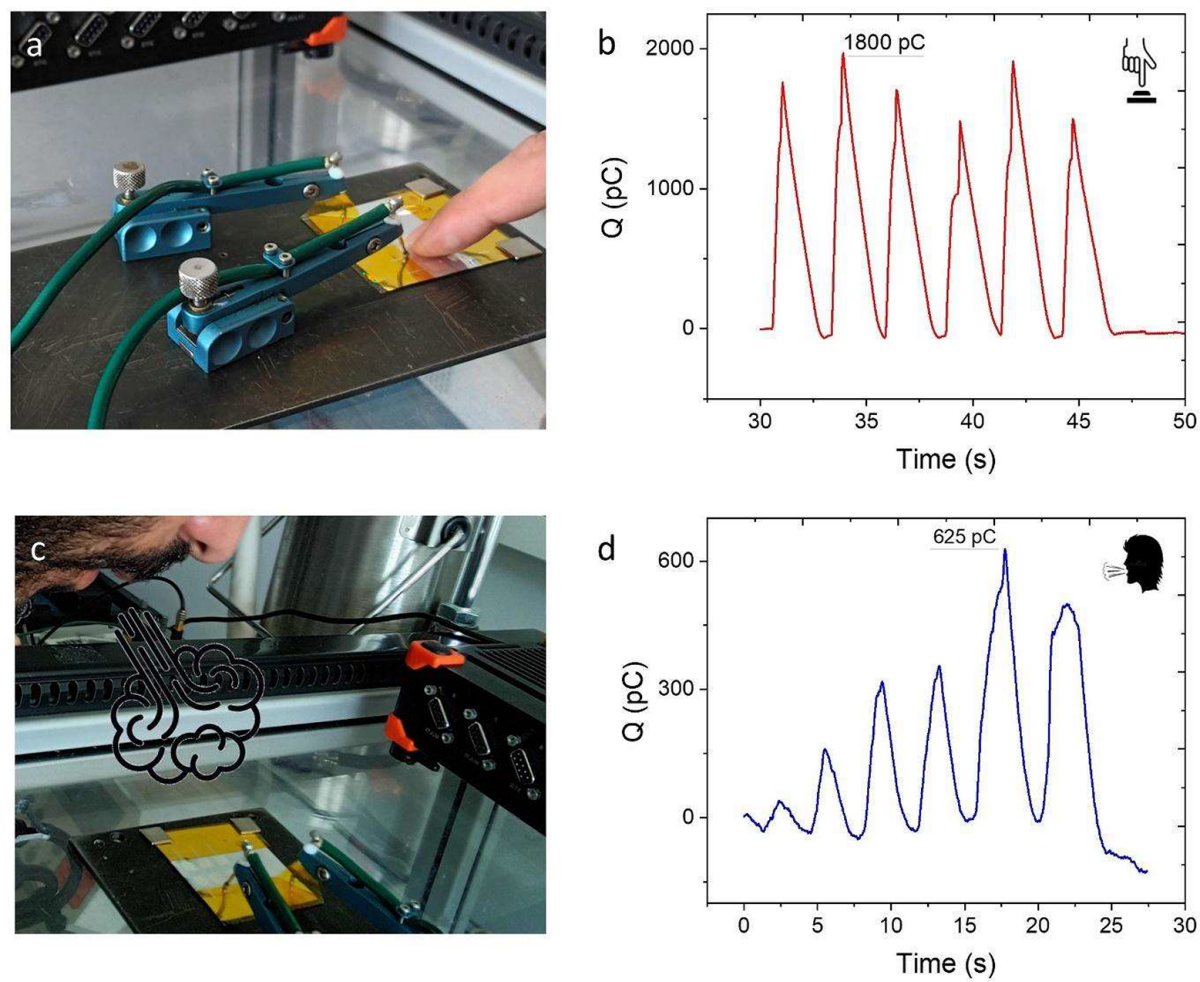
(a,b) Response to six cycles of force excitation through finger touch yielding a maximum response of 1800 pC. (c,d) Response to five cycles of air blown from a human mouth (*Q*_*max*_ = 625 pC).

The devices obtained with the new fabrication method were further tested for touching and breathing. The response of the multistimuli-responsive nanorod skin to six cycles of force excitations from touching is about 1800 pC, as shown in Figure 3a,b. Additionally, five cycles of breath blown from the human mouth are shown in Figure 3c,d. For consecutive blows, a maximum *Q* = 625 pC is measured. Comparing these results to the ones previously obtained with the old fabrication (ref (17)), one can observe that the touch response is much higher now, while the breathing response is similar. This agrees with Figure 2, in which the improvement in mechanical response using the new fabrication is more evident than that upon temperature and humidity stimulation. Probably, the swelling of the hydrogel caused by temperature and humidity stimulation affects the charge transfer at the interface more strongly than the improved adhesion provided by the new fabrication route, hiding its benefits.

### Optimization of the Template Mechanical Properties

In an attempt to explore possible pathways toward the improvement of the device efficiency, we fabricated, with the new method, a device embedded in different templates, one soft with a Young’s modulus of 200 MPa (*E*_*soft*_) and a harder one with a Young’s modulus of 2 GPa (*E*_*hard*_).

**Figure 4:**
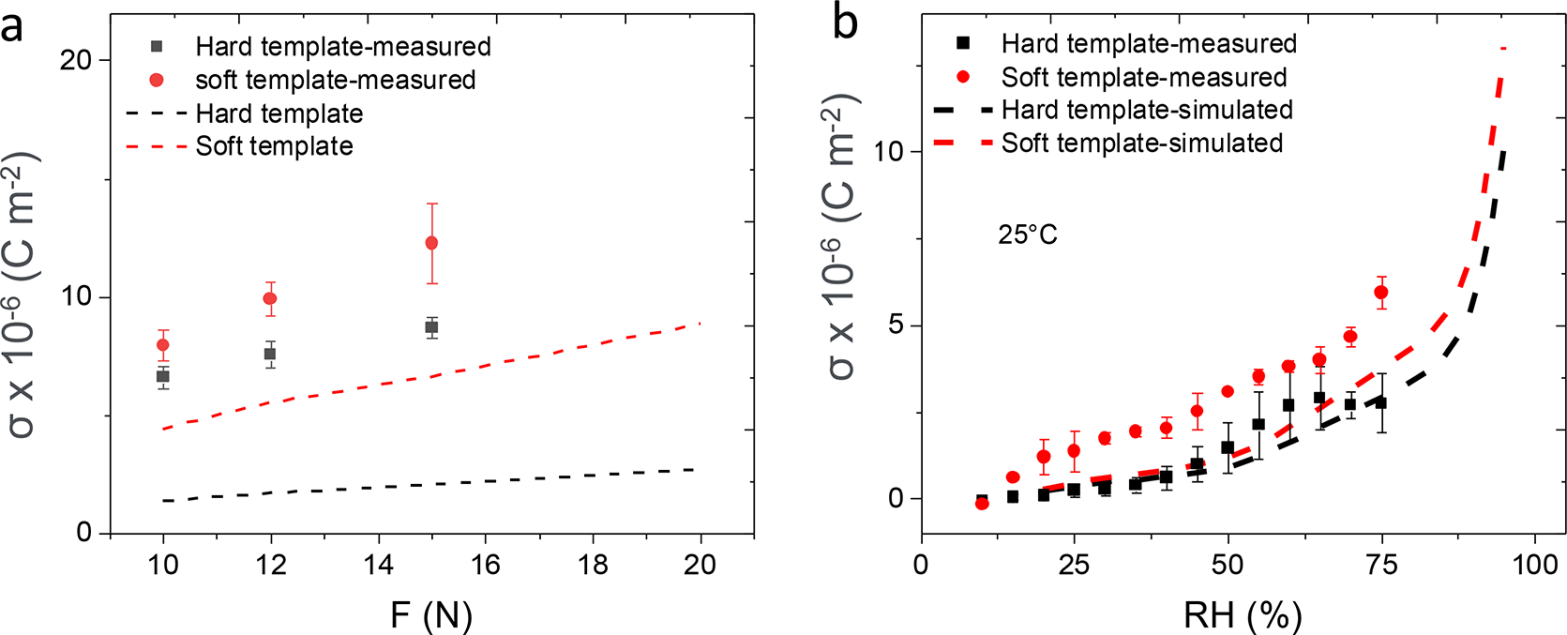
Charge density σ as a function of (a) the force *F* and (b) the RH at 25% for a device fabricated with the new method on two different templates, i.e., a hard one and a soft one. The FEM model (dashed lines) is compared to the experimental data (data points, averaged over three measurements).

Figure 4 shows the charge density σ as a function of *F* and RH, where the influence of the template rigidity is investigated. Simulations were run before the experiments to prove our hypothesis. The experimental data followed what the model predicted. Figure 4a shows that according to the simulation, the use of a softer template should lead to a large improvement in the charge density σ compared to the harder template (2.04 × 10^–6^ for the hard template vs 6.63 × 10^–6^ C m^–2^ for the soft template at *F* = 15 N). Accordingly, the experimental data showed a maximum charge density of σ = 12.2 ± 1.69 × 10^–6^ C m^–2^ at *F* = 15 N for the soft template, whereas for a sample with a hard PUA template only 8.7 ± 0.42 × 10^–6^ C m^–2^ could be observed. The enhanced σ is attributed to the higher deformability of the soft template, which is reflected in higher strain on the ZnO shell, resulting in the generation of more piezoelectric charges. While the simulation shows the enhancement in piezoelectric charge, it fails to reproduce the exact experimental data. Such deviations between the experimental and the simulated curves are attributed to the limits of the model. The model is based on bulk single crystal ZnO with a single preferential orientation along the 002 plane. In reality, instead, our ZnO is polycrystalline with some crystals oriented along the 002 plane and some oriented along the 100 plane. As explained in detail in our previous work, this leads to an increase in the measured piezoelectric charge, compared to the simulated one, because also the deformation along the transversal axis contributes to the signal.^17^

The influence of the PUA template mechanical properties on the response to humidity is shown in Figure 4b, where the use of a soft template enhances the response magnitude with the same trend validated experimentally. Figure S2 illustrates the influence of the template modulus on the temperature response. Here the simulated data also show a higher piezoelectric charge density when a soft template is used.

### Optimization of the Hydrogel Cross-Linker Percentage

In addition to the template mechanical properties, also, the cross-linker (CL) fraction in the hydrogel was varied to try to enhance the sensitivity of the devices; to the previously tested content of 25%, we added also the 35%. This comparison was made on devices fabricated with the new method and using the softer template, i.e., in the conditions previously optimized. At room temperature, hydrogel planar films with CL = 25% show a swelling of ∼225% of their initial thickness (dry state), while films with CL = 35% show ∼200% swelling. In addition, the cross-linker fraction has an influence on the mechanical properties of the hydrogel (*E*_*25%cl*_ = 15 ± 0.5 MPa and *E*_*35%cl*_ = 18 ± 0.5 MPa).^25^ This can influence the applied stress on the ZnO shell.

**Figure 5:**
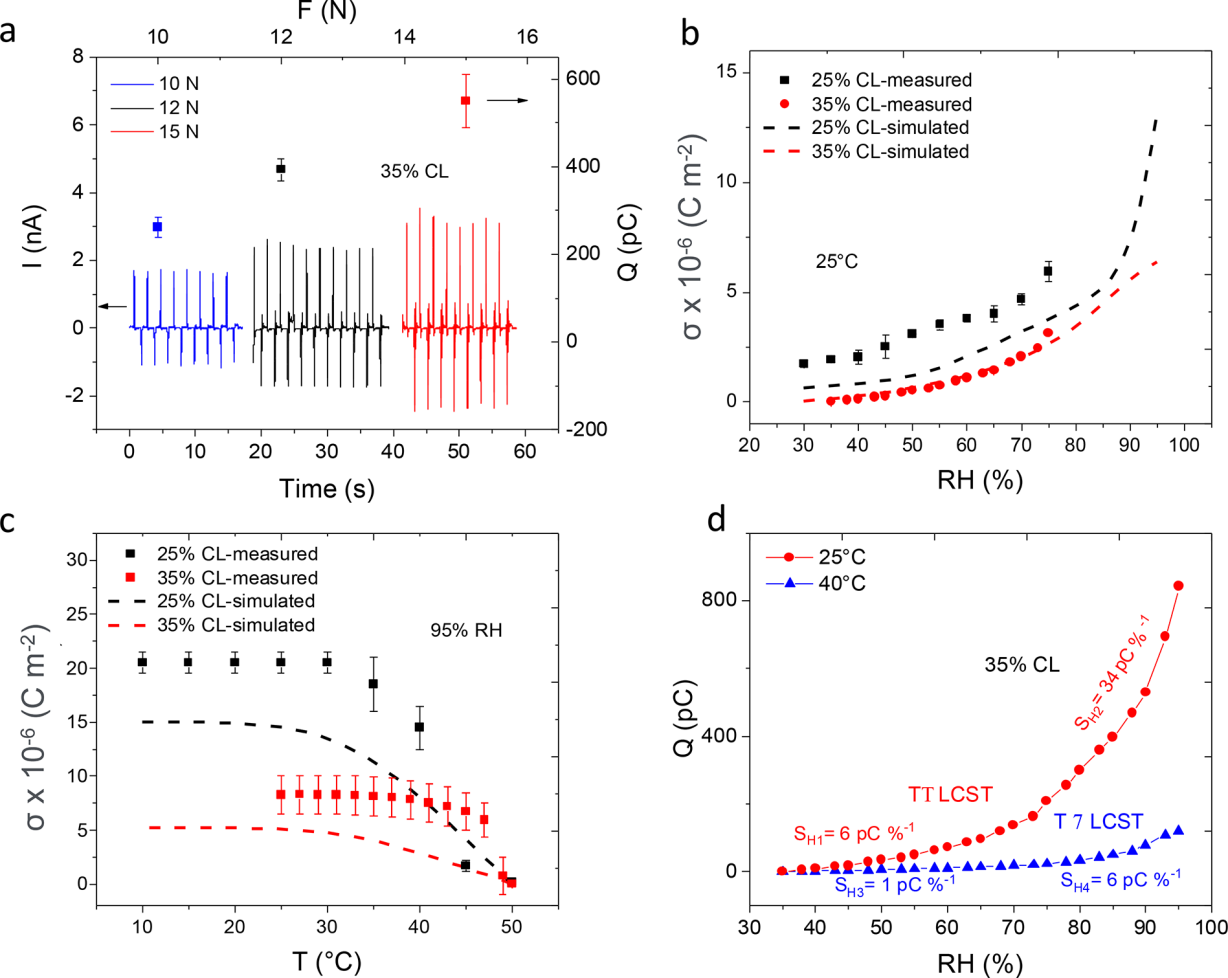
(a) Piezoelectric current *I* (vs time) and charge *Q* (vs force) response to force *F* = 10, 12, and 15 N, over 8 to 10 cycles of press and release (the results are averaged over three electrode fields). Charge density as a function of the (b) relative humidity at 25 °C and (c) of the temperature at 95% RH for devices containing the hydrogel with different cross-linking (CL) percentages. The experimental data are compared with the results from FEM simulations. (d) Charge response to humidity at 25 and 40 °C for the device with 35% CL. The sensitivity *S*_*H*_ is indicated in the figure.

In Figure 5a, the cycles of current corresponding to press and release with a force of 10, 12, and 15 N on a device made with 35% CL hydrogel are shown. It can be observed that the peak-to-peak current of each cycle varies slightly, which is attributed to the hydrogel/Ag interface as well as plastic deformation of the hydrogel layer/core due to contact with the relatively hard stamp. Additionally, the use of a flexible PET substrate promotes mechanical deformation of the active layer, where in-plane and out-of-plane strain components, which are enhanced with increasing force magnitude, influence the micromechanical deformation of the substrate and active layer. The substrate delayed relaxation results in a difference of the current peaks between press and release. This leads to the above-mentioned fluctuations between cycles.^26^

Figure 5b,c shows the results of humidity and temperature stimulation, respectively, for devices built including either a hydrogel with 25% CL or a hydrogel with 35% CL. In both figures, one can see that the hydrogel with lower cross-linking has better performance. This can be attributed to the larger swelling ratio that is measured at lower CL percentages. This assumption is validated by the FEM model, also revealing that a hydrogel with lower cross-linking results in a higher charge density upon humidity and temperature stimulation.

Figure 5d shows the piezoelectric charge *Q* measured on the device made with 35% CL, when changing the humidity at 25 and 40 °C. These measurements were done in an environmental chamber, i.e., on an extended humidity range. The extended humidity range allowed to calculate more precisely the sensitivity to humidity *S*_*H*_: the ratio between the change in charge and the change in RH.
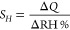
It is obvious that two different *S*_*H*_ values can be calculated, depending on the humidity range. At 25 °C, *S*_*H*_ was 6 pC %^–1^ for the range 35–80% and 34 pC %^–1^ for the RH range 80–95%. At 40 °C, the difference in sensitivities was still measurable even though with a smaller amplitude. Such difference in sensitivity for the different humidity ranges is related to the nonlinear dependence of the hydrogel swelling with humidity.^7^ Since the swelling amplitude is smaller above the LCST (i.e., at 40 °C), the difference in sensitivities is smaller, too. Again, the sensitivities reported in Figure 5d for the hydrogel with 35% cross-linker are lower than the ones derived for devices containing the hydrogel with 25% cross-linker that were reported previously in ref (17).

### Impedance Analysis

While interesting, the piezoelectric measurements have the limitation that the response from force, humidity, and temperature stimulation is not easily distinguishable. Literature shows that many pressure/force, humidity, and temperature sensors rely on change in the active material resistance or capacitance to achieve the desired sensitivity;^26−32^ such parameters can be probed with GEIS, as an alternative and more comprehensive route for signal readout compared to the piezoelectric measurements.

**Figure 6:**
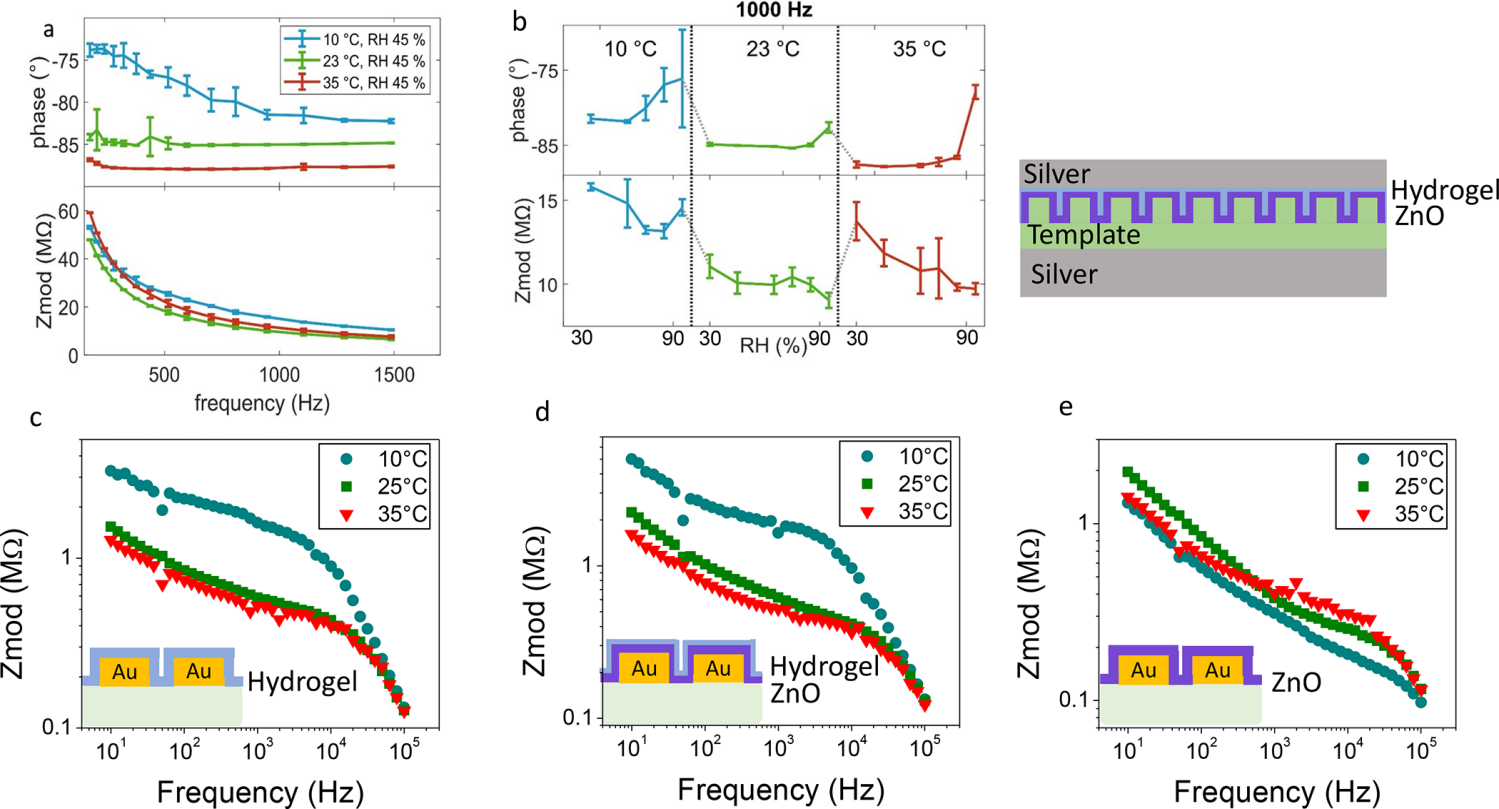
Electrochemical impedance characterization: (a) Bode plots showing the phase and the impedance (*Z*_*mod*_) as a function of frequency *f* = 200–1500 Hz of the devices represented in the scheme on the right exposed to 10, 23, and 35 °C at 45% RH. (b) Phase and *Z*_*mod*_ extracted at 1000 Hz versus the relative humidity (RH = 30–95%) in sequentially performed temperature steps. (c–e) Bode plots of the impedance measured at different *T* in water (100% RH) as a function of the frequency of a hydrogel layer (c), a bilayer ZnO + hydrogel (d), and a ZnO layer (e) deposited on Au electrodes.

The core–shell nanorod devices were sequentially exposed to temperatures of 10, 23, and 35 °C. At each temperature, the humidity was ramped from 30 to 95%. The devices without hydrogel, comprising only the PUA template and the PUA template with ZnO, sandwiched between top and bottom Ag electrode, showed no measurable impedance data, highlighting that the measurable changes in impedance are linked to the presence of the hydrogel. Figure 6a displays the Bode plots at the three temperature steps and 45% RH (exemplary). The impedance (*Z*_*mod*_) decreases exponentially, and the phase increases within the frequency range *f* = 200 to 1500 Hz for all the performed measurements. This is a standard behavior for multilayers composed of dielectric materials, where each layer can be modeled as a parallel capacitive and resistive component (RC element).^33^ A falling *Z*_*mod*_ above the so-called cut-off frequency (dipolar relaxation) is a typical performance of dielectrics.^33−35^ Given that the input voltage and the output current inside a capacitor have a 90° phase shift, the increase in the phase angle with increasing frequency can be explained by the decrease in impedance of the capacitive component of the dielectric. This eases current flow (path of least impedance is favorable).^36^ Moreover, it can be observed that the phase shifts more toward −90° with increasing temperature. At 10 °C, the phase is −80° at *f* = 1000 Hz, while it is −87° at 35 °C. A phase close to −90° is typical of a more capacitive system. Considering the system between the electrodes (hydrogel + ZnO + template) as a parallel plate capacitor, its capacitance *C* is related to the dielectric constant ε by
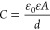
where ε_0_ is the dielectric constant of free space, *A* is the surface area of the multilayer system, and *d* is the total thickness. Upon changes in temperature and humidity, both ε and *d* change in the hydrogel layer: when the hydrogel takes up water, both the thickness and the dielectric constant increase (since at 25 °C the hydrogel has a dielectric constant of ca. 5–6 for frequencies below 10^5^ Hz,^17^ and the one of deionized water is 78.4^37^). This leads to two opposite effects on the capacitance, since *C* is directly proportional to ε but inversely proportional to *d.* At the humidity level *RH* = 45% (related to the data in Figure 6a) the polymer swelling is negligible, since the water that is adsorbed in the hydrogel mostly fills up the existing pores.^7^ Therefore, the changes in the impedance can be attributed mostly to changes in the dielectric constant. Such changes are evident in the phase, but no clear differences can be observed on the *Z*_*mod*_ measured at different temperatures. We think this is because the change in the dielectric constant of the thin hydrogel layer is hidden by the larger dielectric component coming from the template, which represents a thick insulating layer whose ε does not change with temperature.

The phase and *Z*_*mod*_ were extracted at the standard sampling frequency of 1000 Hz, taken as exemplificative, and plotted versus the relative humidity at the sequentially performed temperature steps (Figure 6b). Here one can easily observe that *Z*_*mod*_ decreases with increasing RH as observed also in other humidity sensors.^38,39^ Upon increasing RH, the hydrogel absorbs water molecules in the whole humidity range but swells only above 80% RH and at low temperature, as previously demonstrated.^7^ Therefore, the decrease in *Z*_*mod*_ with increasing RH (in the regime where no swelling is expected) can be explained by an increase in the dielectric constant (due to the presence of water) and thus an increase in capacitance. Only at 10 °C and at RH > 80%, when also the thickness of the hydrogel changes, the *Z*_*mod*_ increases. The water absorption can be probably better followed with the phase shift. At 23 and 35 °C, the phase stays constant up to very high RH, resembling the swelling curve of the hydrogel layer, where the thickness is changing only at very high humidity.^7^

To eliminate the influence of the template and deepen the investigation on the active layers (ZnO and the hydrogel), more GEIS experiments were conducted on a ZnO coating, a single hydrogel coating, and on a bilayer ZnO + hydrogel deposited in direct contact with Au electrodes on printed circuit boards (PCBs). The measurements in humidity showed a decrease in *Z_mod_* with RH, similarly to what is shown in Figure 6b for the full devices.

For observing the effect of the temperature alone, the coatings deposited directly on the Au electrodes were immersed in Milli-Q water baths at 10, 25, and 35 °C. The results are shown in Figure 6c–e. Figure 6c,d show the Bode plots for the PCBs coated with the hydrogel and hydrogel + ZnO, respectively. Here we clearly see that at low frequencies, *Z_mod_* decreases with the temperature. In addition, a clear jump can be observed between 10 and 25 °C. In this case, the changes in thickness are considerable. The corresponding decrease in impedance can be explained considering that the hydrogel goes from a swollen state at 10 °C to a shrunken state at 25 and 35 °C. As a consequence of the thickness decrease, the capacitance increases and the impedance decreases. At high frequencies, resistive effects seem to prevail, and no significant differences can be observed among the different *Z_mod_* curves. Figure 6e shows the Bode plots of the impedance measured on PCBs coated with only ZnO. Here the curves are much closer to each other, and no clear effects of temperature can be evidenced. This is expectable, considering that the thickness of the ZnO does not change in the temperature range of the experiments. The shape of the curves looks also somewhat different in this case, compared to the two-slope behavior of the curves in Figure 6c,d: *Z_mod_* decreases almost with a single slope with the frequency. Such behavior is typical of less porous and more capacitive coatings,^37^ which fits the ZnO characteristics.

**Figure 7:**
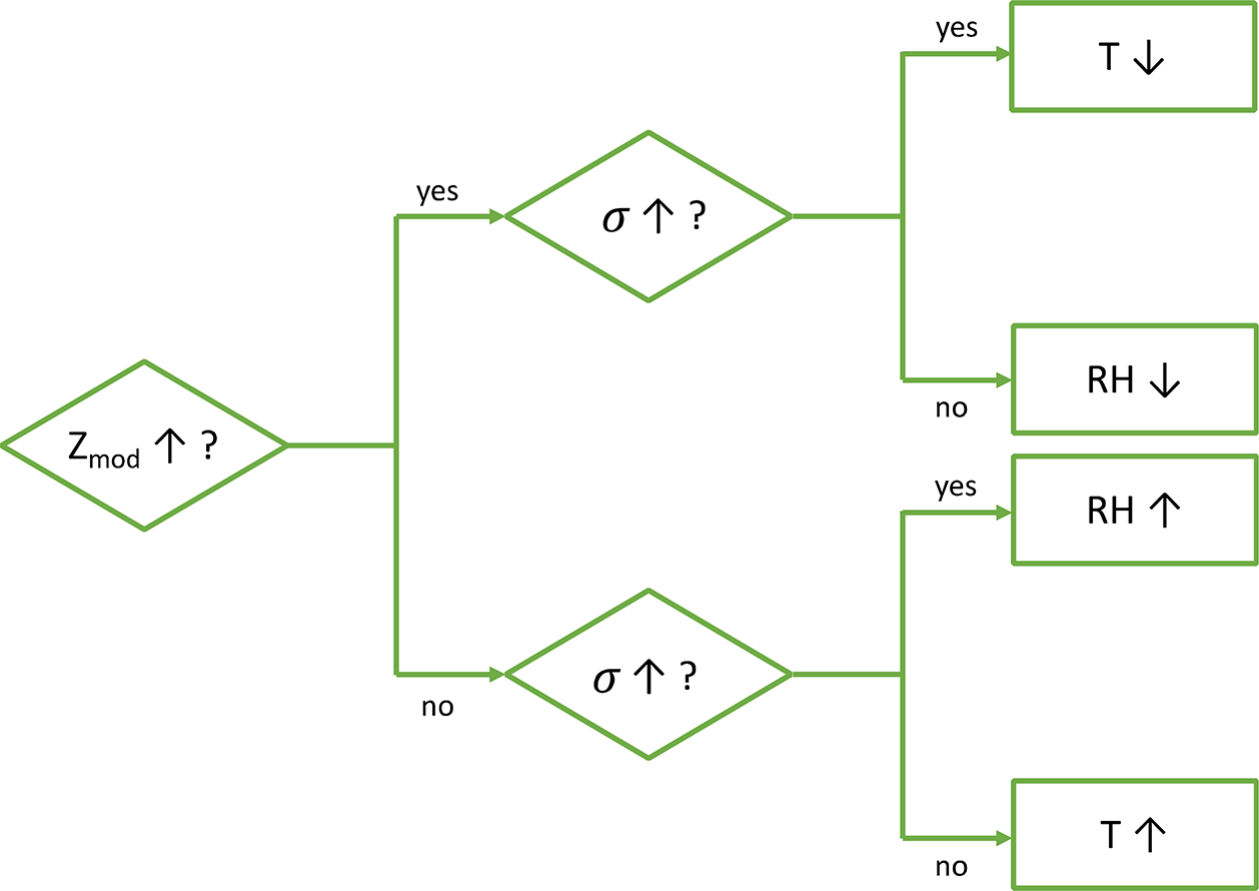
Flowchart showing the effect of temperature and humidity changes on the impedance (*Z_mod_*) and the piezoelectric charge density (σ). This figure shows that a decrease in temperature can be identified by an increase of the impedance and of the piezoelectric charge density, while if they both decrease, it means that the temperature is increasing. When the humidity decreases, the impedance increases, but the piezoelectric charge decreases, and contrarily, an increase in humidity implies a decrease of impedance and increase in piezoelectric charge.

To conclude, the GEIS investigation illustrates that the measurement of the impedance on these systems can be used to gain a deeper understanding of temperature and humidity changes. In addition, the results combined with the piezoelectric charge measurement hint to the possibility of distinguishing between temperature and humidity stimulation, since the impedance increases with decreasing humidity and temperature, whereas the piezoelectric charge increases with increasing humidity and decreasing temperature. Such information can be combined to gain stimuli recognition as suggested in Figure 7.

## Conclusion

In conclusion, we fabricated force-, humidity-, and temperature-responsive sensors based on nanostructured thin films in one-of-a-kind multichamber reactor combining PEALD and iCVD. The sensing performance was optimized with respect to three parameters: (i) the fabrication method that allows in situ interface activation; (ii) the template mechanical properties; and (iii) the hydrogel cross-linker percentage. The multichamber reactor is equipped with a transfer chamber kept under vacuum to permit sample transfer and subsequent depositions without breaking the vacuum. Such a reactor layout reduces surface contaminations arising from transfer processes as well as fabrication time, paving the road for industrial and large-scale fabrication. In addition, it improves the adhesion of the hydrogel to ZnO and reduces delamination related to hydrogel swelling, which yielded devices with better performance. Further, the devices are assessed for multistimuli-responsiveness, with a response of 550 pC to a 15 N force, a maximum sensitivity of 34 pC %^–1^ to humidity, and 200 pC °C^1–^ to temperature. Additionally, FEM simulations as well as experimental measurements indicate an improvement of the performance with the use of a PUA template with a lower Young’s modulus and of a hydrogel with lower cross-linking percentage. Lastly, impedance analysis was employed to further understand and investigate the device’s electrical properties when it is stimulated with humidity and water. The impedance behavior clearly showed that the temperature and humidity have different effects on the capacitance of the system. Stimuli recognition is possible by combining the impedance with piezoelectric charge measurements.

## Supporting Information Available

The Supporting Information provides more details about the FEM simulations used for this study: geometry, equations, swelling data, input parameters. In addition, it shows also how the simulated charge density of devices made with templates with different mechanical properties change with the temperature stimulation (PDF)
