# Supplementary material for: Enhancement of the Sensing Performance of Devices based on Multistimuli-Responsive Hybrid Materials
Source: ACS Appl Mater Interfaces. 2023 Sep 13;16(45):61408–18. doi: 10.1021/acsami.3c08376 (PMC11565566; doi:10.1021/acsami.3c08376)
Supplement: Supplementary file 1 — am3c08376_si_001.pdf [file am3c08376_si_001.pdf]

Supporting Information for:

Enhancement of the sensing performance in multi-stimuli responsive hybrid materials

Taher Abu Ali[1,2]*, Marlene Anzengruber*[1]*, Katrin Unger*[1,3]*, Barbara Stadlober*[2]*, Anna Maria Coclite*[1]*

1 *Graz University of Technology, NAWI Graz, Institute of Solid State Physics, 8010 Graz, Austria*

2 *Joanneum Research Forschungsgesellschaft mbH, MATERIALS – Institute for Surface Technologies and Photonics, 8160 Weiz, Austria*

3 Electronic Sensors, Silicon Austria Labs GmbH, 8010 Graz, Austria

Geometry used in FEM simulations:

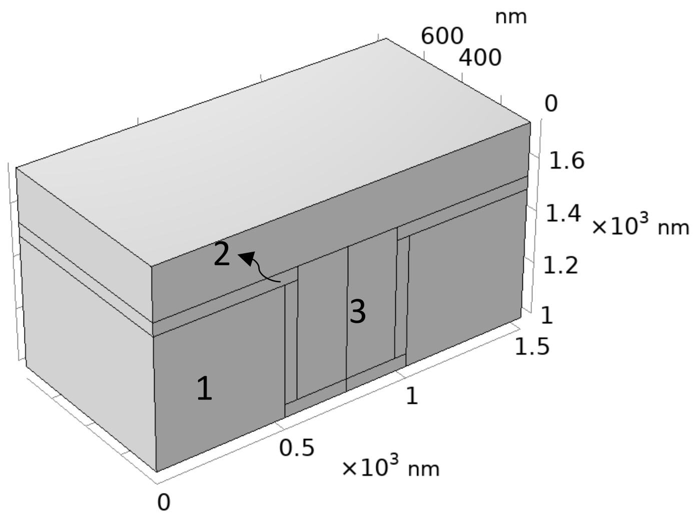

Figure S1. Geometry of half a nanorod used in the FEM model with 1.PUA template, 2.ZnO shell and 3.Hydrogel core.

Equations used in FEM simulations:

Hygroscopic swelling:

$$\varepsilon = \beta \; x \; C_{mo} \qquad (S1)$$

Where,

ε is the hygroscopic strain

β is the hydrogel swelling coefficient (m^3 Kg^{-1})

C_{mo} is the moisture concentration in air (Kg^{-1} m^3), which is equivalent to RH %

Piezoelectric consecutive equations (strain-charge form):

$$\sigma = c_E\varepsilon - eE \qquad (S2)$$

$$D = e\varepsilon + \varepsilon_0\varepsilon_r E \qquad (S3)$$

Where,

σ is the stress (Pa)

c_E is the stiffness matrix (Pa)

ε is the strain

e is the coupling matrix (C m^{-2})

E is the electric field (V m^{-1})

D is the electric field displacement (C m^{-2})

ε_0 is the vacuum permittivity (F m^{-1})

ε_r is the relative permittivity matrix

Charge conservation:

$$\rho_v = \nabla . D \qquad (S4)$$

Where,

ρ_v is the charge density (C m^{-3})

∇ is the divergence (m^{-1})

D is the electric field displacement (C m^{-2})

Material properties used in FEM simulations:

Table S1. Swelling coefficient *β* and moisture concentration in air C_{mo} of p(NVCL-co-DEGDVE) 25% nominally cross-linked at 10, 25, 35 and 50 °C for *RH* = 20 – 95.

RH [%]	C_{mo} (10 °C) [kg m^3]	β (10 °C) [m^3 kg^{-1}]	C_{mo} (25 °C) [kg m^3]	β (25 °C) [m^3 kg^{-1}]	C_{mo} (35 °C) [kg m^3]	β (35 °C) [m^3 kg^{-1}]	C_{mo} (50 °C) [kg m^3]	β (50 °C) [m^3 kg^{-1}]
20	0.002	-	0.005	0.3	0.008	-	0.02	-
30	0.003	-	0.007	0.35	0.012	0.1	0.025	0.1
40	0.004	0.4	0.009	0.4	0.015	0.15	0.033	0.2
50	0.006	1	0.01	0.6	0.02	0.3	0.04	0.3
60	0.0065	2	0.013	1	0.023	0.4	0.05	0.5
70	0.007	4.2	0.016	2.1	0.027	0.7	0.06	0.6
80	0.0075	6.1	0.018	4.1	0.03	1.3	0.066	0.7
90	0.008	26.4	0.02	9.5	0.035	3.7	0.075	1.5
95	0.009	28.6	0.021	10	0.037	4.7	0.08	1.7

Table S2. Swelling coefficient *β* and moisture concentration in air C_{mo} of p(NVCL-co-DEGDVE) 35% nominally cross-linked at 10, 25, 35, 40 and 50 °C for *RH* = 30 – 95 %.

RH [%]	C_{mo} (10 °C) [kg m^3]	β (10 °C) [m^3 kg^{-1}]	C_{mo} (25 °C) [kg m^3]	β (25 °C) [m^3 kg^{-1}]	C_{mo} (35 °C) [kg m^3]	β (35 °C) [m^3 kg^{-1}]	C_{mo} (50 °C) [kg m^3]	β (50 °C) [m^3 kg^{-1}]
30	-	-	0.007	0.17	0.012	-	0.025	0.062
40	0.004	-	0.009	0.64	0.015	0.45	0.033	0.23
50	0.006	0.45	0.01	1.56	0.02	-	0.04	0.46
60	0.0065	1.31	0.013	2.28	0.023	1.42	0.05	0.66
70	0.007	2.42	0.016	3.14	0.027	-	0.06	0.94
80	0.0075	7.62	0.018	4.70	0.03	-	0.066	1.35
90	0.008	19.13	0.02	7.44	0.035	-	0.075	1.94
95	0.009	19.3	0.021	7.96	0.037	-	0.08	2.16

Table S3. Input parameters of different materials used in FEM models.

Component	Description	Symbol	Value
ZnO	Stiffness matrix	c_E	$\begin{pmatrix} 2.1\,x\,10^{11} & 1.2\,x\,10^{11} & 1.05\,x\,10^{11} & 0 & 0 & 0 \\ 1.2\,x\,10^{11} & 2.1\,x\,10^{11} & 1.05\,x\,10^{11} & 0 & 0 & 0 \\ 1.05\,x\,10^{11} & 1.05\,x\,10^{11} & 2.1\,x\,10^{11} & 0 & 0 & 0 \\ 0 & 0 & 0 & 4.2\,x\,10^{10} & 0 & 0 \\ 0 & 0 & 0 & 0 & 4.2\,x\,10^{10} & 0 \\ 0 & 0 & 0 & 0 & 0 & 4.4\,x\,10^{10} \end{pmatrix}$ Pa
	Poisson's ratio	v	0.3
	Density	ρ	5680 Kg m^{-3}
	Coupling matrix	E	$\begin{pmatrix} 0 & 0 & 0 & 0 & -0.48 & 0 \\ 0 & 0 & 0 & -0.48 & 0 & 0 \\ -0.56 & -0.56 & 1.32 & 0 & 0 & 0 \end{pmatrix}$ C m^{-2}
p(NVCL-co-DEGDVE)	Young's modulus	E	12 MPa
	Poisson's ratio	v	0.3
	Density	ρ	1200 Kg m^{-3}
	Relative permittivity	ε_r	2.7
PUA	Young's modulus	E	200 MPa or 2 GPa
	Poisson's ratio	v	0.4
	Density	ρ	1060 Kg m^{-3}
	Relative permittivity	ε_r	4.97

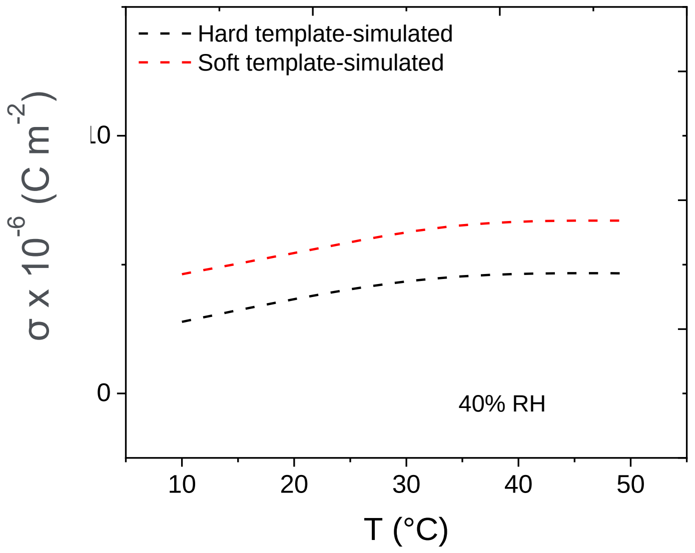

Figure S2: Simulated charge density σ as a function of T (@ 40% RH) when using a soft or hard template to embed the nanorod sensors.
